# The Heart and Artificial Intelligence—How Can We Improve Medicine Without Causing Harm

**DOI:** 10.1007/s11897-023-00606-0

**Published:** 2023-06-09

**Authors:** Christoph Reich, Benjamin Meder

**Affiliations:** 1 Department of Internal Medicine III, Precision Digital Health, University of Heidelberg, Im Neuenheimer Feld 410, 69120 Heidelberg, Germany; 2 Informatics for Life, Heidelberg, Germany; 3 German Center for Cardiovascular Research (DZHK), Heidelberg, Germany; 4 Department of Genetics, Genome Technology Center, Stanford University, Stanford, CA USA

**Keywords:** Digital health, Artificial intelligence, Personalized medicine, Privacy, e-Cardiology

## Abstract

**Purpose of Review:**

The introduction of Artificial Intelligence into the healthcare system offers enormous opportunities for biomedical research, the improvement of patient care, and cost reduction in high-end medicine. Digital concepts and workflows are already playing an increasingly important role in cardiology. The fusion of computer science and medicine offers great transformative potential and enables enormous acceleration processes in cardiovascular medicine.

**Recent Findings:**

As medical data becomes smart, it is also becoming more valuable and vulnerable to malicious actors. In addition, the gap between what is technically possible and what is allowed by privacy legislation is growing. Principles of the General Data Protection Regulation that have been in force since May 2018, such as transparency, purpose limitation, and data minimization, seem to hinder the development and use of Artificial Intelligence.

**Summary:**

Concepts to secure data integrity and incorporate legal and ethical principles can help to avoid the potential risks of digitization and may result in an European leadership in regard to privacy protection and AI. The following review provides an overview of relevant aspects of Artificial Intelligence and Machine Learning, highlights selected applications in cardiology, and discusses central ethical and legal considerations.

## Introduction

Artificial Intelligence (AI) is a generic term that describes the ability of a machine to simulate intelligent behavior. In the past 10 years, significant successes in language processing, object/pattern recognition, and bioinformatics have contributed to the success of AI. Due to the increasing flood of data after the introduction of technologies such as whole-genome sequencing and mobile devices in everyday clinical practice, modern cardiologists will be required to integrate and thus interpret information from a wide range of fields in biomedicine [[Bibr CR1][Bibr CR2]••]. With the help of AI, the cardiologist should be provided with support tools to increase the effectiveness and performance of clinical cardiology. In perspective, the clinician could be freed from routine tasks, so that core competencies such as empathy, attention, and time for physician–patient interaction could be strengthened.

AI systems need to be trained with data to be able to associate similar groups based on characteristics and outcome data. Demographic data, medical reports, laboratory findings, and imaging methods are often used for this purpose. These algorithms require a large amount of training data to be able to adequately answer questions. Machine Learning (ML) as a sub-area of AI includes statistical and mathematical methods, which make it possible to learn patterns and laws based on existing data sets. Deep learning (DL) is in turn a sub-area of ML and is based on artificial simulations of neural networks. It is fascinating that with DL even unstructured and highly complex information—such as images, sounds, or movie sequences—can be processed in a highly efficient way, in real time. Natural Language Processing (NLP) uses unstructured data such as medical reports or articles from medical journals. NLP enables machine processing of natural language. By extracting targeted information, NLP provides structured, machine-readable data, which can then further supply ML algorithms to support diagnosis, treatment planning, and risk assessment [3]. AI algorithms are already transcribing medical dictations with almost no errors. With the speech processing services such as Amazon Comprehend Medical (Amazon, Seattle, WA, USA) precise information such as illness, medication, dosage, strength, and frequency are claimed to be extracted from unstructured sources such as doctor’s notes, reports on clinical studies, and patient files [4]. Text mining in electronic health records is also increasingly being used to support clinical trials by selecting patients who meet inclusion and exclusion criteria [5]. After tidying up large volumes of unstructured text data present in electronic health records, such as demographic information, medical history, and medication, potential participants for clinical trials can be identified, reducing the time and cost associated with manual recruitment processes.

The process of gaining deep information by using AI algorithms is still partly unclear with AI systems. In particular, deep artificial neural networks are still referred to as black-box models because their information processing is not fully understandable for humans, and thus, decisions seem to be made autonomously. This also leads to the fact that black-box models are now recognized by many ML scientists as one of the main obstacles to the use of ML in medicine. Yet not all ML methods represent black-box models. Research groups focusing on “Explainable AI” or “Transparent AI” are looking for ways to better understand hidden logic and individual outputs. It is important that cardiologists confronted with these techniques in clinical decision support or in the interpretation of new research have a critical understanding of both their strengths and limitations [6•].

## COVID-19 as an Accelerator for Clinical AI and Digital Health

The respiratory COronaVirus Disease 2019 (COVID-19) triggered by SARS-CoV-2 (Severe Acute Respiratory Syndrome Coronavirus 2) drastically changed our everyday life with the global outbreak in early 2020. The pandemic has worrying medical, psychological, and socio-economic consequences. Due to the lack of evidence-based treatment options and the lack of vaccines at the beginning of the pandemic, far-reaching measures were introduced to slow down the unrestricted spread of SARS-CoV-2, to avoid overloading hospital infrastructure, and, ultimately, to protect known risk groups. Data on the COVID-19 disease is currently being collected and shared worldwide like never before. AI benefits from the amount of data generated and helps to gain deeper knowledge. Therefore, we need to figure out a well-considered balance between data privacy and public health concerns.

In consideration of the massive amounts of data generated by apps and wearables, AI tools are gaining a central role in data structuring and analysis and therefore are providing more efficient and accurate methods for analyzing and interpreting patient data. With strict lockdowns in place and a heightened fear of exposure to the virus, many patients with cardiovascular diseases and a consequent increased risk of morbidity under COVID-19 have opted to receive virtual care instead of in-person appointments. This shift has pushed healthcare providers to rapidly implement remote monitoring systems, allowing patients to send vital signs, ECG and photoplethysmography readings, and further disease-related data to their doctor from the safety of their own home. This, in turn, allows treating physicians to maintain care delivery and recommend changes in treatment, such as increasing the dosage of heart-failure medication or, in the event of deteriorating health, a recommendation for hospitalization. Patient surveys indicate that telemedicine has several benefits, including increased comfort during virtual appointments (Teleconsultation) and the ability to carry on with work or leisure while receiving medical care [7]. Remote cardiac monitoring has a long tradition in cardiology allowing the exchange of digitized data from implanted or wearable devices especially in the fields of heart failure, atrial fibrillation, and ischemic heart disease [8• 9]. In a concise comment Cowie and Lam indicated that COVID-19 has changed our conversation about the value of remote monitoring [9]. The ubiquitous availability of smartphones and wearables combined with internet connectivity capabilities and advances in sensor technology offers a great chance to fundamentally change outpatient consultation and treatment in a patient-centered manner. New digital measures should specifically promote patient participation and actively involve them in the diagnosis and treatment process. In the future, smart devices and direct-to-consumer technologies will make it easier for “e-patients” to access their own health data, which has so far been stored in data silos and only passed on to them to a limited extent. Smart devices and direct-to-consumer technologies enable not only continuous monitoring and a contribution to patient-centered health data, but also new diagnostic procedures and treatments outside the hospital. Furthermore, telemedicine enhances access to healthcare, especially for individuals residing in rural areas. One example for teleconsultation is the TeleCheck-AF approach, which was set up within an extremely short time at the beginning of the COVID-19 pandemic across different European centers to monitor patients with atrial fibrillation using on-demand photoplethysmography-based heart rate and rhythm monitoring [10•]. Despite the many clearly evident benefits of telemedicine in cardiology, some drawbacks should not be overlooked, including data security risks such as hacking and unauthorized access to patient data, and ethical issues such as inadequate physician–patient interaction, loss of privacy, or barriers to telemedicine in specific patient populations. Finally, further research is clearly needed to evaluate the long-term impact of telemedicine and AI on patient outcomes and the practice of cardiology.

## Artificial Intelligence in Cardiovascular Medicine

**Fig. 1:**
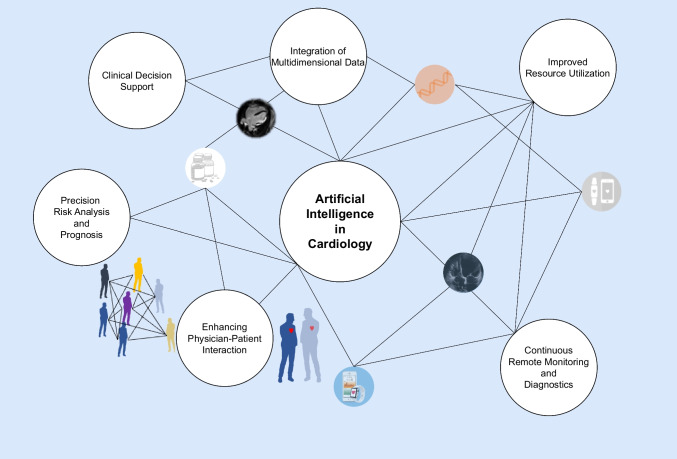
Applications and potential of AI in Cardiology. (1) Clinical Decision Support (CDS) enables integration of all important information for clinical and guideline decisions. CDS is gaining significant importance due to increasing data accumulation; (2) Precision Risk Analysis and Prognosis as well as (3) Integration of Multidimensional Data represent concepts of personalized medicine and can be achieved by applying different domains of AI. They describe the journey from population-based medicine to concepts like patient-like-me; (4) Improved Resource Utilization can result from AI taking over repetitive time-consuming tasks, freeing up physician time for dedicated patient care, and thus (5) enhancing the physician–patient interaction; (6) Continuous Remote Monitoring and Diagnostics includes comprehensive outpatient-centered health care with remote monitoring and teleconsultations as well as mobile devices including associated apps that provide health information to patients, collect a wide variety of physiological parameters, and place the patient at the center of care

AI systems can help to extract additional, often nuanced, information from various already established routine diagnostic tools such as ECG, echocardiography, MRI, CT, cardiac catheterization, biomarker analysis, and genetics. As a result, physicians are liberated from repetitive, time-consuming tasks, allowing them to focus more on their core competencies such as empathy and time for physician–patient interaction. This also translates into improved resource utilization, optimizing the allocation of valuable resources within the healthcare system, ultimately leading to more efficient, patient-centered care. With the development of smartphones, wearables, and the associated apps plenty of new possibilities were added to the asset of smart health data (e.g., to measure pulse frequency and blood pressure, upgrade smartphones to become a stethoscope, ECG device, or measure blood glucose, all at the ambulatory level and more or less continuously) [8•]. The high transformative potential is well illustrated by the existing research results in the field of cardiovascular medicine. Figure 1 shows an overview of the current applications and the potential of AI in cardiovascular medicine that are discussed within this review.

### ECG

As early as 1998, ML found its way into cardiovascular medicine with automated ECG analysis [11•]. Recent study results have shown that astonishing additional information can be extracted from routine ECGs with the help of ML. Zachi Attia and team trained a neural network with apparently normal 12-channel sinus rhythm ECG to detect whether the patient is suffering from silent atrial fibrillation [12•]. They used data from around 180,000 patients and 650,000 sinus rhythm ECGs. It was discussed that AI in patients with paroxysmal atrial fibrillation recognizes electrophysiological changes at the atrial level on the sinus rhythm ECG, which are not detectable by the human eye. Furthermore, deep-learning convolutional neural networks have been trained to detect asymptomatic left ventricular dysfunction, hypertrophic cardiomyopathy as well as patient’s age, sex, and race based on the ECG alone [13•]. These approaches may not be limited to 12-lead ECGs, but it is conceivable that ECG phenotyping will also become possible using single-lead or multilead ECG signals from smart devices. In the future, it will be particularly relevant to generate data from (randomized) prospective studies with patient outcomes in order to validate and verify the use of deep ECG phenotyping and encounter possible challenges and limitations.

### Imaging

The ability of AI to classify and interpret image data is remarkable. AI promises to automate time-consuming, repetitive clinical tasks, reduce examiner-dependent variability, and predict phenotypes that are challenging or not apparent to the human eye in the first place. In March 2020, Ouyang et al. reported their AI algorithm (EchoNet-Dynamic) to be able to classify left ventricular ejection fraction using echocardiographic recordings [14]. The algorithm was trained on more than 10,000 videos using a 3D DL-model. Particularly noteworthy is the detection and integration of both spatial and temporal changes in the left ventricular borders. Seah et al. used neural networks to automatically detect radiological patterns of heart failure [15]. It should be emphasized here that the authors succeeded in directly visualizing the features that were necessary for their classification. In the segmentation of cine MRI images of the left ventricle, Ngo and team were able to surpass manual segmentation using DL [16]. Thus, the use of AI algorithms saves medical doctors valuable time and enables improved evaluation of radiological images. In 2016, the British computer scientist Geoffrey Hinton, who is best known for his contributions to artificial neural networks, said: “It's just completely obvious that in five years DL is going to do better than radiologists” [17]. This statement refers to automated pattern recognition. Clinicians and radiologists cannot be replaced by AI since they are needed for the integration and interpretation of clinical data as well as for the interaction between patients and colleagues [18].

Arterys (Arterys, San Francisco, CA, USA) uses DL and cloud computing, which is storage space available over the Internet, for the automatic analysis of cardiac MRI images. Time-consuming routinely performed analysis can be accelerated and automated [19]. In addition, advanced methods, such as quantifying the entire delayed enhancement in the left ventricle, enable a more detailed observation and analysis of cardiac MRI sequences. With the “AI Rad Companion,” Siemens Healthineers (Siemens Healthineers, Malvern, PA, USA) have also set themselves the goal of replacing repetitive, time-consuming tasks with AI. The Butterfly iQ (Butterfly Network, Guilford, CT, USA) is a new tool for bedside diagnostics, which has already been approved by the FDA (US Food and Drug Administration). In contrast to conventional ultrasound transducers, which generate ultrasound waves employing piezoelectric quartz or ceramic vibrators, a silicon chip is used here. The app supplied interprets the resulting ultrasound images using AI [20].

### Risk Analysis, Prognosis, and Personalized Treatment

AI-powered clinical decision support systems (CDSS) provide physicians with a *virtual medical coach* that uses comprehensive input from an individual, which is deep learned to enhance diagnostic accuracy, optimize treatment strategies, and improve individual patient outcomes [21]. AI-powered CDSS can process vast amounts of complex data, including patient demographics, clinical histories, genomic data, and imaging results. By identifying patterns and correlations within this data, AI can empower clinicians with actionable insights to guide their decision-making. Lately, Tayal et al. presented a novel precision-phenotyping approach to subclassify dilated cardiomyopathy into three distinct subtypes [22]. They applied ML on multidimensional data (demographic and clinical features, ECG and CMR data, sequences from 169 genes and 276 proteomic biomarkers) to gain new insights into dilated cardiomyopathy. Whether the classifications represent different phenotypes or different stages of the disease remains unclear. Nevertheless, deep classification in patients with dilated cardiomyopathy will enable tailored personalized diagnoses and thus more targeted therapies to be selected in the future. Identifying more accurate patterns of systolic and diastolic dysfunction in heart failure via a data-driven approach to phenotyping has the potential to improve risk assessment and treatment optimization especially within this heterogeneous condition. The current classification of heart failure, which is based on measurement of the left ventricular ejection fraction, is a practical but oversimplified, and there is growing evidence that diastolic dysfunction is composed of multiple subgroups with different patterns of disease manifestation and underlying causes, requiring individualized approaches [23-25]. Particularly now that the successful large-scale randomized trials of SGLT2 inhibitors are opening up new therapeutic options, the use of ML could allow treatment recommendations to be tailored to more refined phenogroups in the future. Another application is the risk stratification of ventricular arrhythmias in hypertrophic cardiomyopathy. Nevertheless, the results are not yet sufficient for clinical introduction [26]. In a study by Alaa et al., the cardiovascular risk of asymptomatic patients was analyzed using data from more than 400,000 patients and over 450 variables [27]. This study identified new cardiovascular risk factors and reported a superior risk stratification compared to the Framingham Score. Clinical validation must be achieved through follow-up studies, preferably randomized controlled trials. Nevertheless, the existing studies demonstrate the breakthrough potential of improved prognostic applications through ML in cardiovascular medicine.

Digital health assumes easy access to advanced technologies such as recording digital biomarkers using advanced sensor technology or even whole-genome sequencing. This therefore makes the patient the point-of-care and allows him to actively participate in the diagnosis and treatment process. However, as the use of digital health and the collection of personal data continues to expand, it is crucial to consider the ethical principles and personal privacy risks involved. Concepts to ensure data integrity and incorporate legal and ethical principles are clearly needed to avoid the potential risks of digitization and will be discussed in the following section.

## Ethical Principles and Legal Framework for Data Protection

### Responsibility

The use of AI, which can combine almost all possible datasets, and which is even able to generate natural looking images de novo, gives rise to considerable privacy risks and can potentially lead to job losses, discrimination, or social isolation [28••, 29••]. Particular attention must be paid to ensure responsible and ethical handling and use of data under the premise to protect individual’s freedom, personal rights, and autonomy. To turn it around: compliance with ethical norms and principles must not be violated when developing or using AI; it must be strictly enforced. Personal data must be processed with the greatest care. The fear of losing human decision-making sovereignty through fully automated systems can be controlled by adhering to moral limits and a thorough examination. Risks of storage, transfer, and use must be considered at an early stage. In April 2019, the European Commission published its ethics guidelines for a trustworthy AI use, which are based on the Charter of Fundamental Rights of the European Union [30]. The guidelines are based on principles such as data protection and transparency. Social scientists have been able to show how imperceptibly these ethical principles can be violated. An example recently reported by Obermeyer et al. in Science concerns racial bias encoded by ML from observational health care data [31]. Black patients with the same risk score as White patients assessed by a widely used algorithm tended to be much sicker due to racial bias by design and choice of ground truth. The tool was designed to predict the cost of care as proxy for health needs and underestimated severity of disease in Black patients because of unequal access to care and less money spent on care for Black patients. This type of bias is particularly harmful because it can arise from reasonable choices. Encouragingly, the authors reported that this bias can be compensated for. Through careful selection, we can exploit the benefits of algorithmic predictions while minimizing their risks [31]. Potential hurdles to a final AI tool can be addressed through the appropriate selection of ideal data sources, thoughtful interpretation, validation, and generalization of results, and a pervasive evaluation of safety and ethical concerns. In accordance with the ethical and moral principles described by Beauchamp and Childress in their book *Principles of Biomedical Ethics* in 1977, doctors are expected to use new technologies sensibly and only for the benefit of the patient [32]. The four basic moral principles include *respect for autonomy (includes the right to refuse a treatment), non-maleficence (“primum non nocere”), beneficence (act in the best interest of the patient)*, and *justice*. Every new, revolutionary technology can raise new ethical and legal questions; nevertheless, our fundamental values should not be questioned. Table 1 shows relevant aspects from an ethical and legal perspective based on the concepts of the EURAT project group on “*Ethical and Legal Aspects of whole genome sequencing of the human genome*” [33].

**Table 1:** Aspects for regulations relating to collection, saving, usage, transfer, and publication of data records at the institutional level

Responsibility	All participating scientists and institutions have to handle the data in a responsible manner. Competences are to be assigned to all parties involved to prevent conflicts and diffusion of responsibilities
Transparency	The methods used must be described and explained fully and made understandable to the public
Data protection and data security	Data must not be lost and must be protected from unauthorized access with measures that cannot be bypassed
Accessibility of data	The data are to be made available to the scientific community to the greatest extent possible, on one hand, to enable the best possible use of the data, on the other hand for the scientific verifiability of results (quality of research)
Data avoidance and purpose limitation	Only those data may be gathered, saved, and forwarded that serve the specific research purpose

### Transparency

Clinical systems require strict control and supervision. There is a low tolerance threshold, particularly with regard to technical errors. Transparency and efficiency will have a major impact on medical confidence in the use of AI. A large area of research is currently focusing on explaining the so-called black-box models to address clinicians’ and users’ need for transparency. Current explanatory techniques have their limitations (e.g., approximation of high-dimensional nonlinear models by linear models), which cardiologists and cardiovascular researchers need to be aware of in order to make informed decisions about when and how to use them [6•]. Transforming the healthcare system supported by the enormous potential of AI requires a multidisciplinary approach with the involvement of AI developers/users, doctors, ethics workforce, and humanities scholars. This also requires an expansion of the current study plans with a focus on data science and AI products and services.

### Data Protection and Data Security

There is a relevant risk of re-identification due to the powerful analysis algorithms and the enormous amount of data. Already established methods such as pseudonymization and anonymization, which are used specifically to prevent re-identification, can thus become ineffective. Rocher, Hendrickx, and de Montjoye were able to correctly re-identify 99.98% of Americans in each data set using 15 demographic attributes [34]. If sufficiently large amounts of data are used, there is insufficient anonymity in the context of medical data, and it is inevitable that personal or person-related data will be worked with. Nevertheless, when using data acquired from daily life, mechanisms such as *differential privacy* allow to protect individuals within aggregated data but also within individual-level data by introducing random noise that is enough to protect against de-identification but does not harm the purpose to train algorithms. Current and next-generation methods for federated, secure, and privacy-preserving AI include *decentralized, distributed systems* that allow the data to remain with its owner (containers). Centralized solutions (e.g., cloud computing) present several concerns such as increased data volume and issues about data ownership, confidentiality, privacy, security, and the creation of data monopolies that favor data aggregators [35, 36]. Another development in decentralized learning is what is known as Swarm Learning [37]. Unlike federated learning, in which the data also remains local, but model parameters are exchanged centrally, there is no central command center in Swarm Learning. A Swarm network consist of Swarm edge nodes that exchange parameters for learning cooperatively based on rules recorded in a blockchain. Further concepts to preserve confidentiality, privacy and ethics include *cryptography* (e.g., homomorphic encryption), and *secure multi-party computation* (*SMPC*) which prevents single parties to retrieve the entire data on their own [35]. Yao’s protocol, a cryptographic method, has been successfully applied in the field of genetic sequencing and diagnostics and proved to preserve participant privacy in up to 99.7% [38].

### Accessibility of Data, Data Avoidance, and Purpose Limitation

A key prerequisite for AI application is the availability and quality of data. Medical AI algorithms must learn from curated patient data to later work accurately and trustworthy for the single patient. On the other hand, we must ask ourselves how we can protect vulnerable patient data at a point, where patient data becomes an increasingly valuable commodity and the line between innovation and exploitation dissolves. Since the early 1990s, the development of data protection law has been based on an increase in the volume of data and a growing need for privacy protection. The principles of data protection established with the EU GDPR include *transparency, lawfulness* and *fairness, purpose limitation, data minimization, accuracy, storage limitation* as well as *integrity* and *confidentiality* as stated in Chapter II Art 5 [39]. The primary goal is to protect personal data. However, AI is particularly characterized by the use and processing of big data. If AI algorithms are trained on relatively small datasets, this will often result in limited robustness and potentially leads to biased results for unspecified subgroups. According to the GDPR’s *data minimization requirement*, a trend from *Big Data* to *Smart Data* can be observed using exactly the data that is required to answer a specific question. The principle of *purpose limitation* also plays a major role here: AI tries to establish hidden relationships. When using existing large amounts of data, they are decoupled from the purposes they originally served. Ideally, the data that needs to be used to train AI systems originate from sources that guarantee privacy by design and consent. Some companies, for instance, do not buy available data to develop their algorithms in smart devices, but conduct smaller or larger trials on their own or together with academic partners, to collect the necessary information with written consent from the participants. While this approach is expensive and more laborious, the data often has “clinical trial quality” and can address many aspects needed to ensure provenance and quality of the measured data items from the beginning and leading to secure and ethical use of patient data. The *PROMISE (Personal medical safe)* study, for instance, evaluated within a controlled clinical trial new methods that allow patients to decide for themselves on the access to their genetic data [40•]. The genetic data is encrypted and securely stored on servers. Innovative cryptographic processes such as functional encryption are used to only analyze very defined parts of the stored gene sequences. Access is only granted after a specific, one-time authorization from the patient. It can thus make an important contribution to self-participation of patients in the digital health era.

## Conclusion

The transition toward digital medicine has been substantially accelerated over the last few years. With the tremendous progress, we risk falling behind in regulating and monitoring the emerging technologies. Therefore, ethical and legal confrontation is of utter importance. Patient empowerment and consent should be emphasized more strongly in the regulatory process, and increasingly sophisticated methods for privacy-preserving and explainable AI are required while minimizing the cost to innovation. Improving access to healthcare through outpatient-generated data can help to allocate resources and decentralize patient care. This can be of great benefit to public health in the treatment of widespread and common diseases like coronary heart disease, chronic heart failure, or, of topical interest, pandemics like COVID-19. Physicians are taking on new role in advising patients on digital health, which implies a duty to educate themselves on the safe and beneficial use of digital technologies. Thorough patient education and transparent handling of patient-related data are the basis for paving the way further for digital medicine.

## Abbreviations

AI: Artificial Intelligence
CDC: US Centers for Disease Control and Prevention
CDSS: Clinical Decision Support System
COVID-19: COronaVirus Disease 2019
DL: Deep learning
FDA: US Food and Drug Administration
GDPR: General Data Protection Regulation
ML: Machine Learning
NLP: Natural language processing
SARS-CoV-2: Severe acute respiratory syndrome coronavirus 2
